# A Real-Time EMG-Based Fixed-Bandwidth Frequency-Domain Embedded System for Robotic Hand

**DOI:** 10.3389/fnbot.2022.880073

**Published:** 2022-06-30

**Authors:** Biao Chen, Chaoyang Chen, Jie Hu, Thomas Nguyen, Jin Qi, Banghua Yang, Dawei Chen, Yousef Alshahrani, Yang Zhou, Andrew Tsai, Todd Frush, Henry Goitz

**Affiliations:** ^1^State Key Laboratory of Mechanical System and Vibration, Shanghai Jiao Tong University, Shanghai, China; ^2^Department of Biomedical Engineering, Wayne State University, Detroit, MI, United States; ^3^Orthopaedic Surgery and Sports Medicine, Detroit Medical Center, Detroit, MI, United States; ^4^Research Center of Brain Computer Engineering, School of Mechatronic Engineering and Automation, Shanghai University, Shanghai, China; ^5^Prosthetics and Assistive Devices Department, Taibah University, Medina, Saudi Arabia

**Keywords:** short-time fourier transform, real-time control, robotic hand, embedded system, frequency domain, fixed bandwidth, myoelectric signal

## Abstract

The signals from electromyography (EMG) have been used for volitional control of robotic assistive devices with the challenges of performance improvement. Currently, the most common method of EMG signal processing for robot control is RMS (root mean square)-based algorithm, but system performance accuracy can be affected by noise or artifacts. This study hypothesized that the frequency bandwidths of noise and artifacts are beyond the main EMG signal frequency bandwidth, hence the fixed-bandwidth frequency-domain signal processing methods can filter off the noise and artifacts only by processing the main frequency bandwidth of EMG signals for robot control. The purpose of this study was to develop a cost-effective embedded system and short-time Fourier transform (STFT) method for an EMG-controlled robotic hand. Healthy volunteers were recruited in this study to identify the optimal myoelectric signal frequency bandwidth of muscle contractions. The STFT embedded system was developed using the STM32 microcontroller unit (MCU). The performance of the STFT embedded system was compared with RMS embedded system. The results showed that the optimal myoelectric signal frequency band responding to muscle contractions was between 60 and 80 Hz. The STFT embedded system was more stable than the RMS embedded system in detecting muscle contraction. Onsite calibration was required for RMS embedded system. The average accuracy of the STFT embedded system is 91.55%. This study presents a novel approach for developing a cost-effective and less complex embedded myoelectric signal processing system for robot control.

## Introduction

Developing an intuitive control system for assistive robot motions has become a hot research topic recently. In past decades, research has shown that an intuitive control mechanism can improve the exoskeleton performance and an individual's experience (Lotze et al., 2003; Gui et al., 2017). The signal from electromyography (EMG) (myoelectric signals) is a promising physiological signal to reflect motion intention, which comprises the sum of the electrical signals generated by the active motor units (MUs), and EMG signal has been widely used in rehabilitation therapy (Kawase et al., 2017; Yao et al., 2018; Gui et al., 2019; Gordleeva et al., 2020; Li et al., 2021). There are still challenges in processing EMG signals for robot control, including removing systematic noise and artifacts (McCool et al., 2014; Roland et al., 2019), increasing signal-noise ratio (SNR) using special electrode (Zhang et al., 2019; Chen et al., 2020; Fu et al., 2020), determining the onset and offset of muscle contraction (Xu et al., 2012; Yin et al., 2020), and lowering firmware and device cost. Less complex algorithms and firmware are required to develop an optimal signal processing and control system.

Myoelectric-based control systems have achieved high accuracy in laboratory environments or testing offline. However, the real-time usability does not meet the expectations of amputees or patients (Parajuli et al., 2019). Onsite calibration is required for the robotic system to achieve better sensitivity and accuracy of the system performance. One of the key issues for real-time myoelectric control is that control techniques lack robustness for various subjects due to individual bio-variability, particularly in the current embedded systems. The voltage trigger threshold needs to be adjusted among different users. With a lower triggering threshold, unwanted robot motion can be triggered by noise or artifacts, while the user needs to contract muscles harder to let the robot move when a high threshold is set up. Further improvement is required to have a user-friendly system.

A case was reported (Secciani et al., 2019) to demonstrate an EMG-based strategy for the motion control of wearable assistive hand exoskeleton systems. An EMG-based embedded system was developed using a cost-effective microcontroller unit (MCU), the Arduino Nano board. One EMG sensor was placed on the flexor digitorum and another sensor was placed on the extensor digitorum. A patient with spinal muscular atrophy (SMA) type II used the proposed system to grasp 10 differently shaped items. After 1 week of training, the patient was able to complete the task, but the grasping time was longer than the healthy subjects. This paper did not address the variability of different users and related performance accuracy among other users. The system response time was slow with an average grasping time longer than 10 s.

EMG RMS-based time-domain features have been used as an approach to classify nine wrist-hand movements (Raurale et al., 2019). The Myo Armband was used to collect eight channels of EMG signals from the subject's upper forearm. EMG signals were processed by a small set of time-domain features, including integrated-EMG, the natural logarithm of variance, and Root Sum Square. Kernel Fisher's discriminant feature projection (DFP) and radial bias functional neural network (RBF-NN) were used to classify the different movements. The whole system was deployed using ARM Cortex-A53 MCU, and the processing time meets the requirement of real-time usability. Although the proposed system showed high classification accuracy within less processing time, the system needed complex training for different subjects. Complex algorithms are required to process eight-channel EMG signal inputs. It is successful in laboratory research, but no commercial products are available in the market.

EMG signal has been used to control upper arm prosthesis, the DEKA arm using EMG-pattern-recognition (EMG-PR) techniques (Resnik et al., 2018). Twelve upper limb amputees were recruited for the user's experience experiment. Qualitative data were collected through survey questions and interviews. The results showed that most participants preferred the controls of traditional prosthesis rather than the EMG-PR-controlled DEKA arms. However, most participants were positive about the future potential of the EMG-PR-based controlling system. This suggested that improvements are still required in myo-electricity-based control systems.

To date, most of the research reported in the literature using the EMG-based control system is still being performed in laboratory settings. There is a gap between research and product commercialization and mass production. Cost-effective, small, and simple products are preferred by users and clinical applications. EMG-based signal processing system for robot motion control is still not perfect with many challenges including the need for the removal of artifacts and noise. RMS (root mean square) of EMG is a commonly used feature for robot control but can be significantly influenced by motion artifacts and noise (Ho et al., 2011; Salvietti et al., 2016). RMS-based time-domain signal processing includes both myoelectric signals and noise artifact signals, hence, this can yield a large magnitude variance of recorded signals for processing. We hypothesize that the frequencies of artifacts and noise are not within the frequency bandwidth of muscle contraction, hence, the EMG signals associated with muscle contraction can be separated from the whole signal spectrum using a fixed frequency bandwidth methodology, thus directly denoising the signals and reducing the variance of signal magnitude, generating stable signals for robot motion control. This study hypothesized that the frequency bandwidth of noise and artifacts is beyond the main EMG signal frequency bandwidth, hence, the fixed-bandwidth frequency-domain signal processing methods can filter off noise and artifacts only by processing the main frequency bandwidth of EMG signals for robot control. The purpose of this study was to develop a cost-effective embedded system and short-time Fourier transform (STFT) method for an EMG-controlled robotic hand.

Frequency-domain features and short-time Fourier transforms (STFT) have been proposed and used for spectral analysis of EMG signals (Englehart et al., 2001; Phinyomark et al., 2018). However, the frequency-domain EMG processing method has not been used in any commercialized products. The frequency-domain EMG processing has been implemented only in a laboratory setting using a large computer and advanced software, such as MATLAB or LabView software. And most of these previous studies used machine learning (ML) algorithms for frequency-domain EMG signal processing (Da Silva et al., 2008; Larivière et al., 2008; Camata et al., 2010; Costa et al., 2010; Dantas et al., 2010) and they were conducted by performing offline data analysis for conceptualization proof rather than real-time signal processing for robot motion control. Artificial intelligence and machine learning (ML) are emerging techniques in EMG signal processing for motion pattern recognition and robot control. However, the ML-based firmware is expensive and significant efforts are needed for algorithm development (Jiang et al., 2020; Zhou et al., 2021). In recent years, deep learning-based human-robot interaction (HRI) was developed (Qi et al., 2021, 2022; Su et al., 2021a) and achieved a higher recognition accuracy and faster inference speed with the help of GPU. To our knowledge, no cost-effective MCU has been adopted to process EMG signals for real-time robot motion control using the fixed-bandwidth frequency-domain STFT processing method.

The purpose of this study was to develop an EMG-based fixed-bandwidth frequency-domain embedded system using a cost-inexpensive, small-size microcontroller unit (MCU) for volitional robot movement control. Experiments were conducted to identify the optimal myoelectric signal frequency bandwidth responding to muscle contraction. The feasibility of using cost-effective firmware and less complex algorithms were developed and validated among healthy volunteers. The effectiveness of STFT-based myoelectric signal processing was compared with the RMS-based myoelectric signal processing method to determine a better myoelectric signal processing approach.

## Materials and Methods

### Electrode and Locations for EMG Signal Acquisition

This system was designed to control the robotic hand for precise wrist flexion and extension motion and resting control. This motion is one degree of freedom (DoF) in an adaptive manner, and the wrist motion started and stopped at any time and at any position according to the user's intents. The multiple DoFs motion control system was not developed in this study. The flexor carpi ulnaris and extensor carpi radialis longus were chosen as the EMG signal sources for the system. All procedures have been approved by the ethics committee (Institutional Review Board) of Wayne State University.

The electrodes for this system were general-purpose electrodes (BIOPAC, System, Inc.), which had Ag/AgCl contact (11 mm of diameter) and standard snap connection. One channel of EMG signal used three electrodes: two electrodes placed on the targeted muscle and one placed on the olecranon as the ground electrode.

### EMG Signal Processing

The raw EMG signals were rectified and integrated by the MyoWave™ EMG sensor module (SparkFun Electronics, AT-04-001) before being further processed by the STM32 microcontroller unit (MCU) for STFT processing. The rectified and integrated EMG signals (RIEMG) as a time-domain feature can be calculated using the following equation:


(1) $$\begin{array}{l} RIEMG=\int_{t}^{t+T}|x\left(t\right)|dt \end{array}$$

Where *x(t)* is an input EMG signal and ***T*** is the length of the time window.

Root mean square (RMS) was used for time-domain feature extraction, which was calculated as the following equations:


(2) $$\begin{array}{l} RMS=\sqrt{\frac{1}{N}\overset{N-1}{\underset{i=0}{\sum }}x^{2}\left(i\right)} \end{array}$$

Where ***x*** is an input EMG signal, ***N*** is the number of elements of a time window, and ***i*** is the time interval of the integral.

As for frequency-domain features, median frequency (FMED) is the most common feature. FMED is the frequency representing the midpoint of the power distribution in the Power Spectral Density (PSD) and it is the frequency below and above which lies 50% of the total power in the EMG. It is calculated as follows:


(3) $$\begin{array}{l} \int_{0}^{FMED}P\left(f\right)df=\frac{1}{2}\int_{0}^{\infty }P\left(f\right)df \end{array}$$

Where ***P(f)*** is the signal power spectral density function.

The mean frequency (FMEAN) of a spectrum is calculated as the sum of the product of the spectrogram intensity (in dB) and the frequency, divided by the total sum of spectrogram intensity.


(4) $$\begin{array}{l} FMEAN=\frac{\sum_{i=0}^{n}I_{i}\cdot f_{i}}{\sum_{i=0}^{n}I_{i}} \end{array}$$

Where ***n*** is the number of frequency bins in the spectrum, ***f***_***i***_ is the spectrum frequency at bin ***i***, and ***I***_***i***_ is the intensity of the spectrum at bin ***i***.

Short-time Fourier transform (STFT), a time-frequency-domain analysis method was used to determine the main frequency band in responding to muscle contraction. The STFT divided a longer time signal into shorter segments of equal length and then computed the Fourier transform separately on each shorter segment. The equation of STFT is shown as the following:


(5) $$\begin{array}{l} G_{f}\left(\epsilon ,u\right)=\int EMG\left(t\right)g\left(t-u\right)e^{j\epsilon t}dt \end{array}$$

Where ***g(t-u)*** is the window function and *EMG(t)* is the raw EMG signal.

### Integrated EMG Signal Processing and Stepper Motor Controlling System

**Figure 1:**
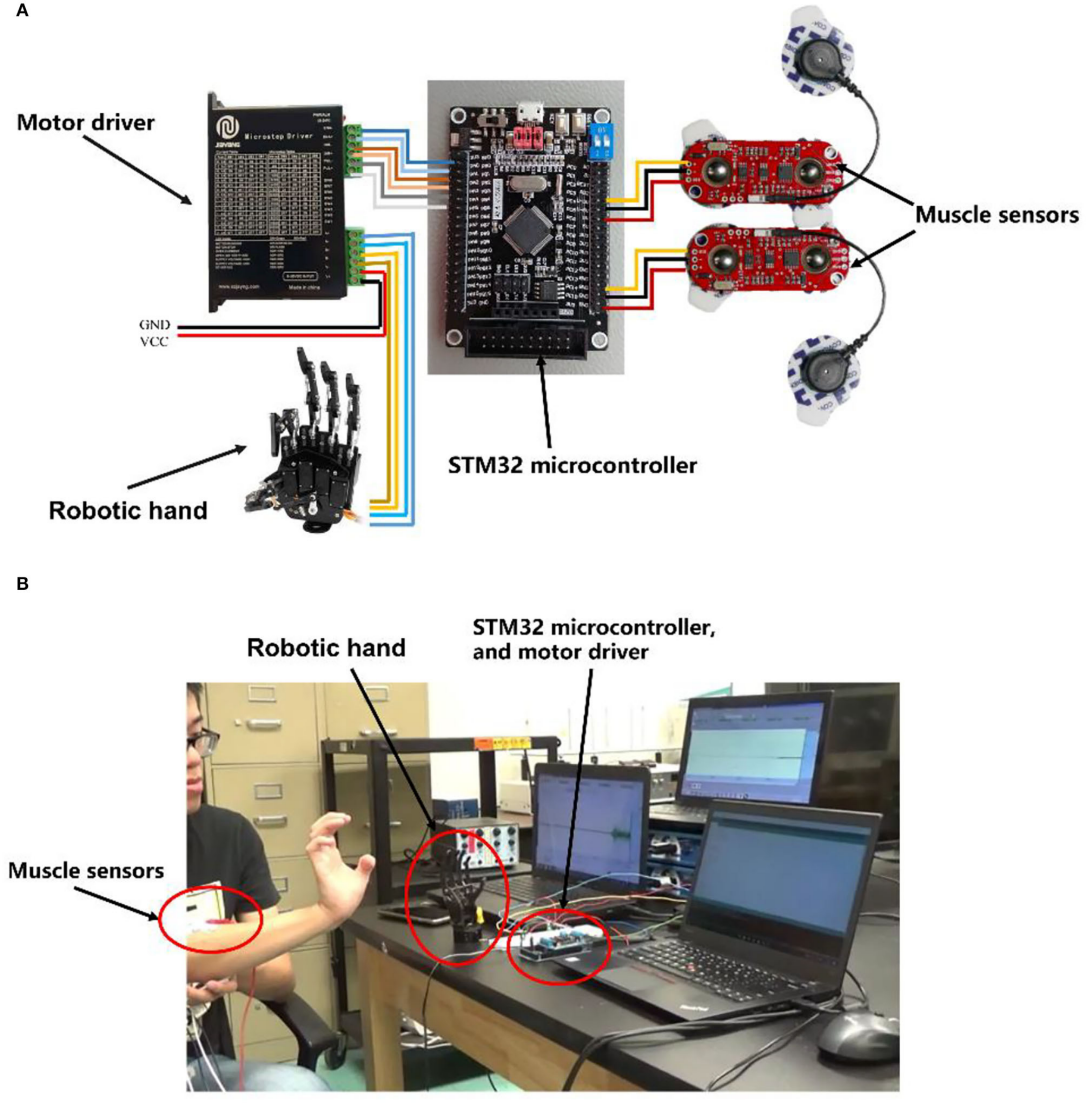
**(A)** Electronics connection schematics of embedded stepper motor controlling system. **(B)** The actual integration and realization of the system.

The system consisted of two MyoWare™ Muscle Sensors (SparkFun Electronics, AT-04-001), one STM32 microcontroller, one stepper motor driver, and one robotic hand. The MyoWare™ module was used to collect, amplify, and filter the signals. The EMG signals were sent to an STM32 microcontroller unit (MCU) for STFT processing, motion pattern recognition, and motor motion control (Figure 1). In the experiment, the robotic hand was controlled by the motor driver and had one degree of freedom, which can perform the open and close motions.

### Algorithm Design for Embedded EMG Signal Processing and DC Motor Motion Control

#### STFT Algorithm and Firmware for Motor Control

**Figure 2:**
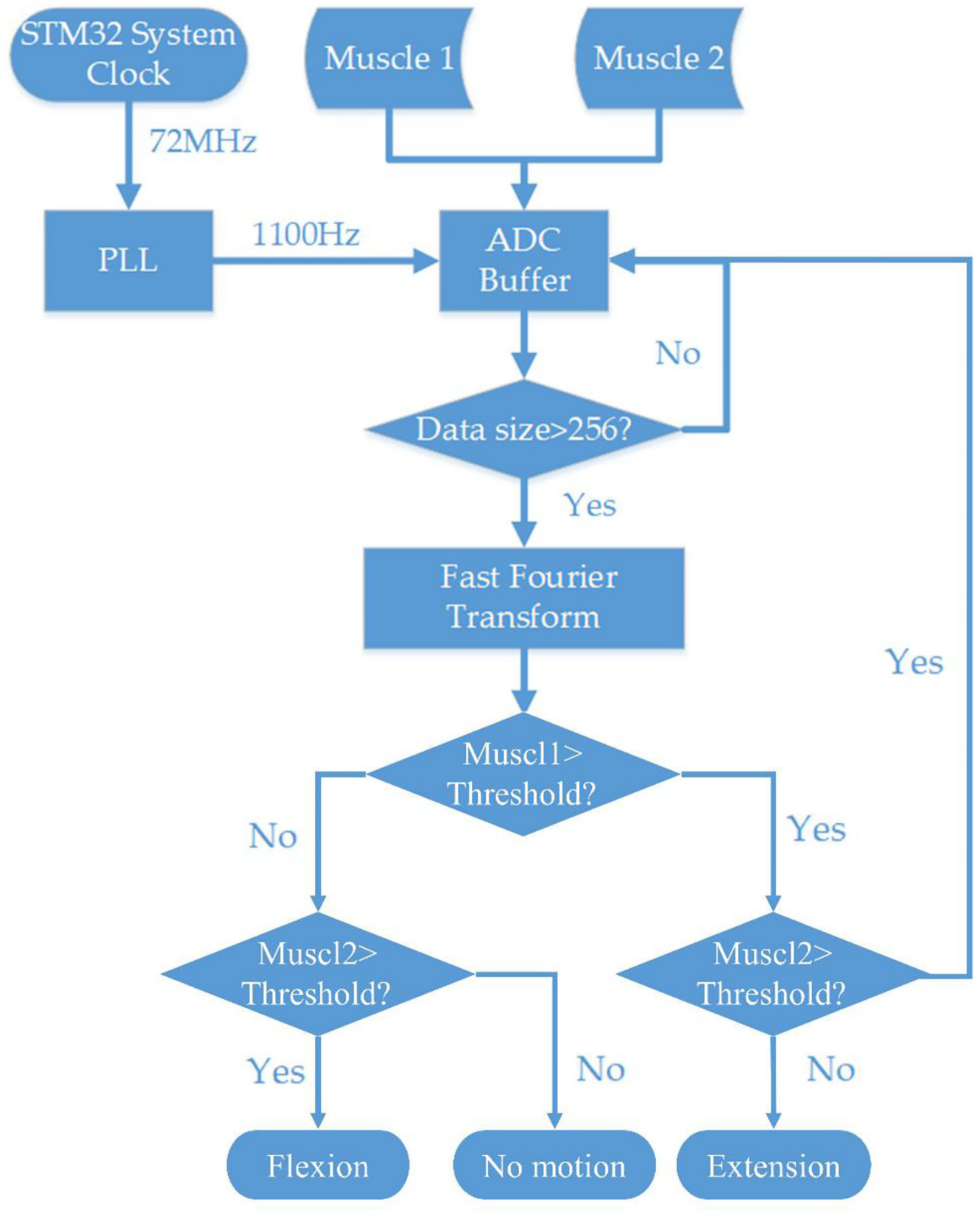
Flowchart of the systematic block diagram of the embedded EMG processing system and stepper motor controlling system.

The STM32 MCU was used to build the embedded system. STM32 has an ARM 32-bit Cortex™-M3 CPU with 128 KB flash memory, and the MCU can operate at a 72-MHz frequency. The sample rate of this system's Analog to Digital Converter (ADC) was defined by the system clock and phase-locked loops (PLL). Nyquist–Shannon sampling theorem described that a sufficient sampling rate should be >2B Hz (B is the band limit of a given bandwidth, representing the maximum frequency of the signals). Because the main power spectrum of EMG signal ranges within the frequency band of 0–500 Hz, the ADC sample rate was set at 1,100 Hz. After the data were collected from the EMG sensor, a 256-point FFT algorithm was applied to obtain the frequency spectrum. STM32 and software clearly showed the magnitude changes of different frequency bands by STFT including the bandwidth responding to muscle contraction. Since the most prominent bandwidth was 60–80 Hz as shown in Figure 5H, algorithms were encoded to monitor the magnitude of 60–80 Hz frequency bandwidth for motion classification. The controlling strategy was set up according to the frequency spectrum and voltage magnitude. The motor motion triggering threshold was set at 50% of the maximum voltage amplitude of the frequency band of 60–80 Hz. Then, the MCU sent the controlling signal through the GPIO pin to the microstep drivers (MB450A) to drive the DC motor. All the controlling algorithms and MCU initialization were written in MDK-Keil software (uVision4) using C++ language (Figure 2).

#### RMS Algorithm and Firmware for Motor Control

The data from EMG sensor were processed using RMS algorithms and STM32 firmware. The triggering threshold was set at the 50% level of amplitude of EMG voltage processed by RMS algorithms indicated in Equation (2). The user's EMG signal was recorded before the triggering threshold was set up to determine the triggering voltage. When the voltage was higher than the threshold, the MCU sent a controlling signal through the GPIO pin to the microstep drivers (MB450A) to drive the DC motor. All the controlling algorithms and MCU initialization were written in MDK-Keil software (uVision4) using C++ language.

### Determination of Optimal Frequency Bandwidth for Motor Control

**Figure 3:**
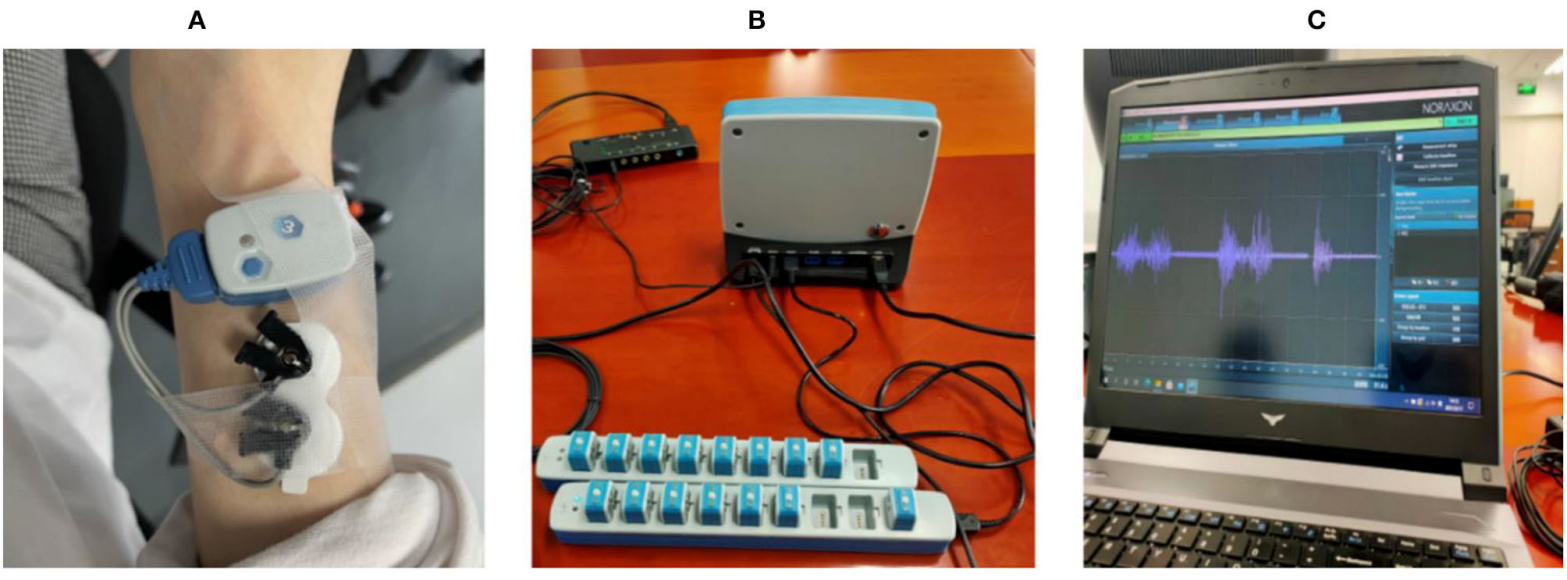
**(A)** shows electrodes placed on the skin surface of the extensor carpi radialis longus muscle; **(B)** shows the wireless EMG acquisition system and its base unit; **(C)** shows that MR3 software and raw myoelectric signals respond to muscle contraction from electromyography.

The purpose of the EMG feature selection experiment was to choose the most appropriate EMG feature for embedded systems. The investigated EMG features included median frequency, mean frequency, RMS of EMG voltage, and the frequency bandwidth responding to contraction. Five healthy subjects (three healthy males, two healthy females, ages 23–27) were recruited for the surface EMG signal recording to determine the EMG characteristics of muscle contraction during wrist motions. Before recording, all subjects were informed in detail about the experiment and the precautions. The EMG signals were recorded using the Noraxon wireless EMG recording system (Noraxon Inc. AZ, USA, sampling rate 2,000 Hz) and Noraxon MR3 software (version 3.12.70. Noraxon Inc. AZ, USA) (Figure 3).

The EMG sensor was placed on the extensor carpi radialis longus muscle to record the EMG signal. Each subject sat on a chair and flexed or extended the wrist with a 10-lb dumbbell held in the subject's hand. Subjects were required to control the time and rhythm for each wrist motion according to the metronome (4 s for one movement) under a 10-lb load. Each subject performed a total of 20 consecutive wrist flexion and extension motions. Then, the subjects rested for 15 min and then performed another 20 consecutive wrist flexion and extension motions. The purpose was to determine if the prolonged repeated muscle contraction led to EMG feature changes.

The recorded EMG data were processed offline using MATLAB R2020a (Natick, Massachusetts: The MathWorks Inc) to measure median frequency, mean frequency, RMS of EMG voltage, and the frequency bandwidth responding to contraction. The obtained RMS data and the frequency bandwidth responding to contraction were then used for the motion control experiment.

### Performance Validation of Embedded EMG-Controlled Robotic System

Six subjects (five healthy males, one healthy female, ages 23–36) participated in this study. It should be noted that there was no overlap between participants in the experiment described in sections 2.5 and 2.6. The purpose of recruiting new subjects was to verify the adaptability of the system to different subjects. Two surface EMG sensors were placed on the forearm and recorded EMG signals were sent to the MCU system to control exoskeleton joint motions. One electrode was placed on the flexor carpi radialis longus muscle and another electrode was placed on the flexor carpi ulnaris muscle.

A pre-training was conducted for each subject. After properly placing the EMG sensors on the muscles, the subjects were asked to freely contract the extensor carpi radialis longus muscle and flexor carpi ulnaris muscle. The DC motor motion triggering threshold for time-domain feature (RMS magnitude) and frequency domain feature (magnitude of a frequency band) was set for each subject.

After finishing the pre-training process, all the subjects were asked to do two sets of standard motions. First, the time-domain feature was used for motion control, and the threshold of the time-domain feature was determined by 50% of the RMS magnitude of the subject. Each subject performed wrist flexion for 20 repetitions and extension for 20 repetitions, and the subjects had a 5-min rest before starting the second set of experiments. The second set of experiments used the frequency-domain feature for robotic hand motion control, and the threshold of the frequency-domain features was determined by 50% of EMG voltage magnitude of bandwidth between 60 and 80 Hz of the subject. Each subject performed wrist flexion and extension 20 times in the same manner. The accuracy of the robotic hand performance was recorded.

The accuracy of the system operation was recorded. When the robotic motion matched the subject's hand motion, the operation was counted as a correct operation. Otherwise, it was counted as an incorrect operation.

### Statistical Analysis

Statistical analysis was performed with SPSS statistical software version 26 (IBM, Armonk, NY, USA). The *t*-test was used to determine the difference in voltage magnitude of EMG signals recorded between two time periods (the first 20-hand operation and the second 20-hand operation) to determine if signal magnitude fluctuated over time in an individual. ANOVA with *post-hoc* LSD was used to determine the difference in measured EMG features between different subjects and the individual variability. Chi-square with Pearson test was used to determine the statistical difference level of the performance accuracy between two different control mechanisms. Statistical significance was defined as a *P*-value smaller than 0.05.

## Results

### Characterizations of EMG Signals Responding to Muscle Contraction

**Figure 4:**
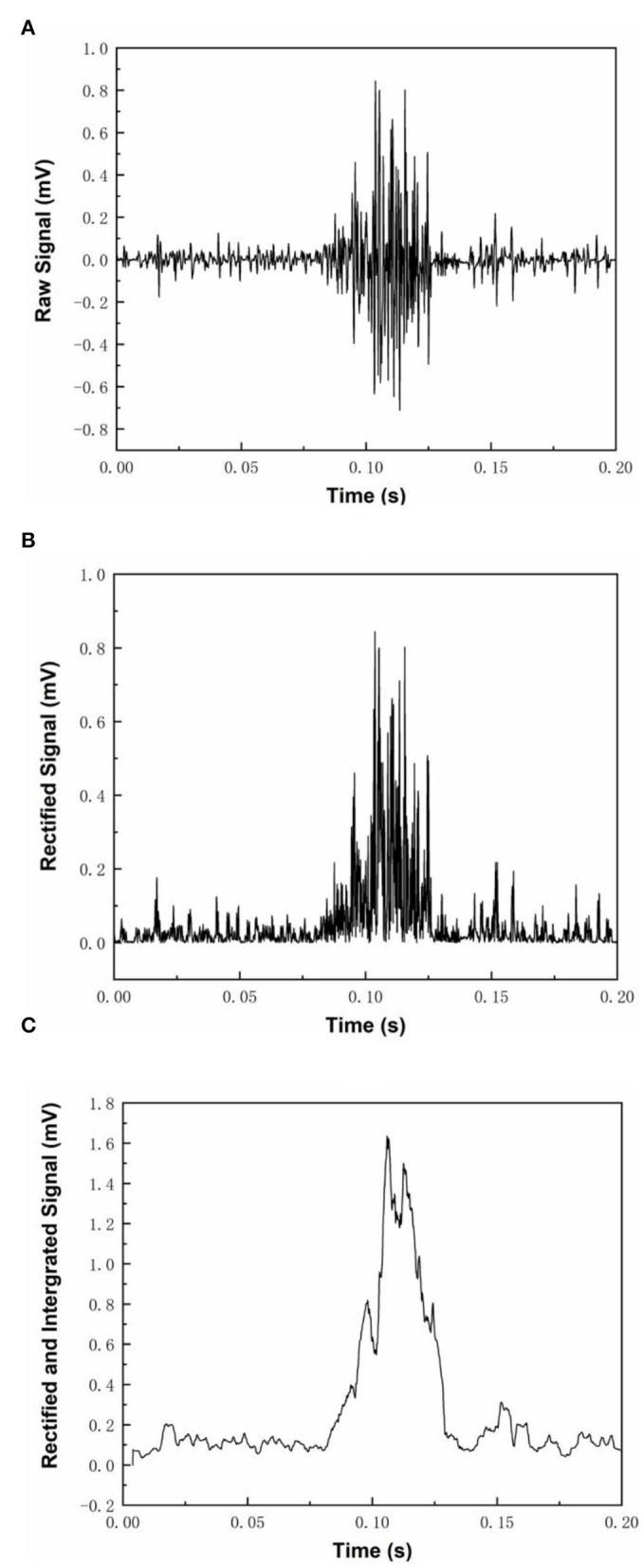
Three different modes of MyoWare™ Muscle Sensor EMG signal output. **(A)** shows the raw EMG signal during muscle contraction, **(B)** shows the rectified EMG signals, and **(C)** shows the rectified and integrated EMG signal.

MyoWare^TM^ module recorded the EMG signal in response to a muscle contraction (Figure 4A). The mean RMS of baseline EMG signal at resting was close to 0 volts of EMG signals could be rectified into positive readings by the MyoWare^TM^ sensors with positive spikes in the signals (Figure 4B). Rectified EMG signals could be integrated and turned into stable curves with fluctuation in amplitude (Figure 4C). The amplitude of the EMG voltage responding to a muscle contraction was high enough to set a DC motor triggering threshold at the 50% level of the magnitude (Figure 4C).

When a subject performed a wrist flexion, the EMG signal from flexor carpi ulnaris became strong while the EMG signal from extensor carpi radialis longus remained at a lower level. On the other hand, when a subject performed a wrist extension, the EMG signal from extensor carpi radialis longus became very strong while the EMG signal from flexor carpi ulnaris returned close to the baseline level. The distinct features were then further processed for robotic hand motion control.

### Characterization of EMG Frequency Band

**Figure 5:**
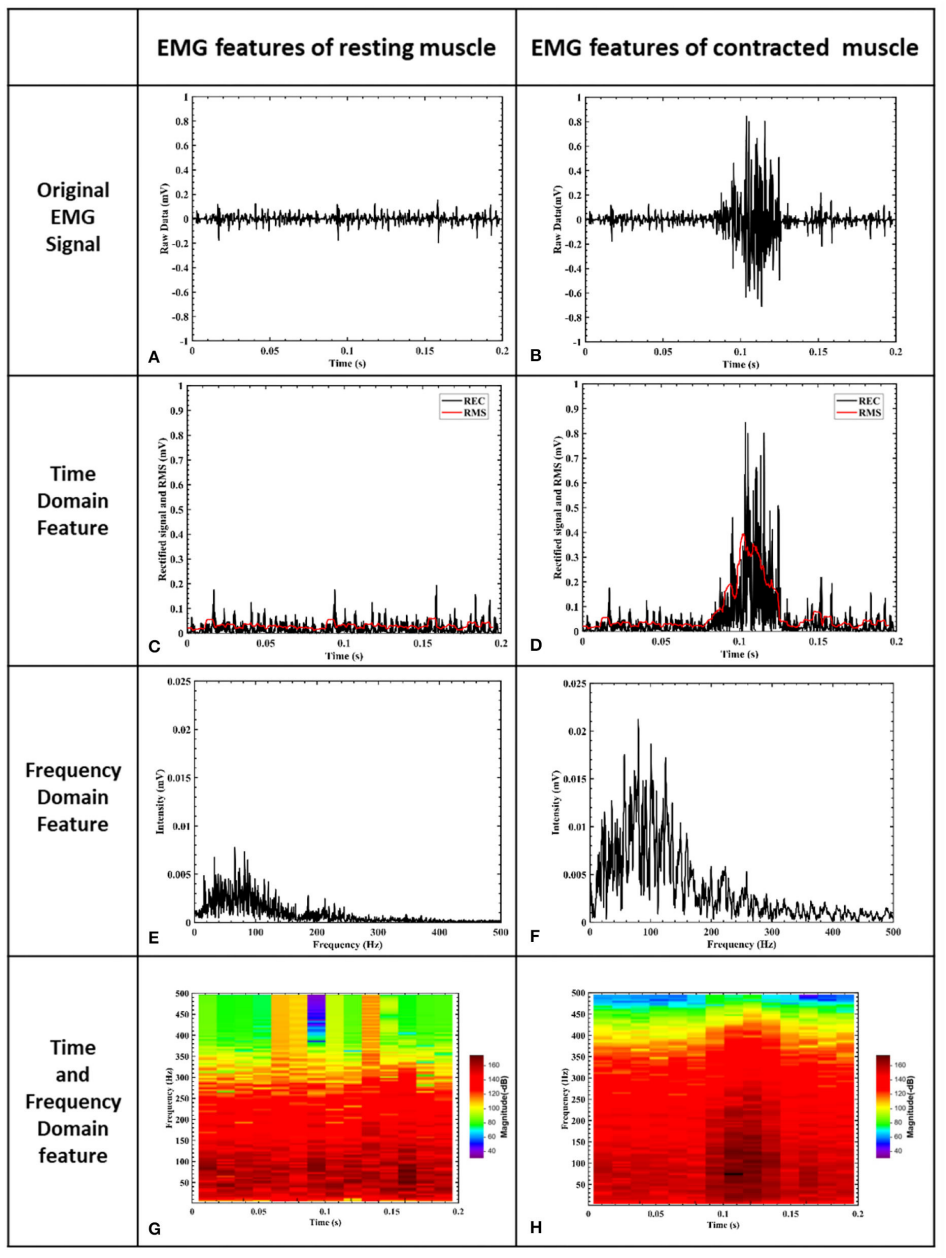
EMG signal features of muscle. **(A)** is the EMG signal filtered by a 10–500 Hz band-pass filter. The x-axis represents time and the y-axis is the corresponding signal amplitude after normalization. **(B)** shows raw EMG voltage amplitude during a muscle contraction. **(C)** shows rectified baseline EMG activity. **(D)** shows rectified EMG voltage amplitude during a muscle contraction. **(E)** shows the frequency domain of resting muscle activity after STFT processing, and the frequency resolution is 5 Hz. **(F)** shows the frequency domain of muscle contraction after STFT processing. The responding bandwidth of muscle contraction is between 30 and 200 Hz, and the frequency resolution is 5 Hz. **(G)** shows the time and frequency domains of EMG signals at resting status, and the frequency resolution is 4 Hz. **(H)** illustrates the time and frequency domains of EMG signals at muscle contraction, and the frequency resolution is 4 Hz. The responding bandwidth of muscle contraction was between 30 and 200 Hz **(F)** and the most prominent was between 60 and 80 Hz with a peak value around 70 Hz (dark red) **(H)**.

Figure 5 shows the results of one single wrist flexion motion using the Noraxon wireless EMG recording system and Noraxon MR3 software. The muscle contraction yielded a clear EMG response (Figure 5B) compared with the resting status (Figure 5A). The power spectrum analysis of the EMG signal frequency demonstrated that the responding bandwidth was between 30 and 200 Hz (Figure 5F) and the most prominent was between 60 and 80 Hz with a peak value around 70 Hz (dark red) (Figure 5H). The spectrogram of STFT is shown in Figures 5E,F. The muscle has a mild baseline activity at resting states (Figure 5A) with a resting frequency band between 20 and 200 Hz similar to the frequency band upon muscle contraction (Figure 5E). At the resting status, EMG signal amplitude showed fluctuation over time as shown in the time-domain window (Figures 5A,C,G). In terms of the complexity of computational algorithms among the four features, the filtered EMG RMS magnitude signal was the simplest but onsite calibration for manual adjustment of the triggering threshold was frequently required to maintain it at a 50% magnitude level. Although the STFT was the most complicated feature, onsite calibration for the adjustment of triggering threshold level was required much less than the RMS-based algorithm. The magnitude of the frequency band between 60 and 80 Hz had less variability and was relatively stable.

### Individual Variability of EMG RMS and Frequency

**Figure 6:**
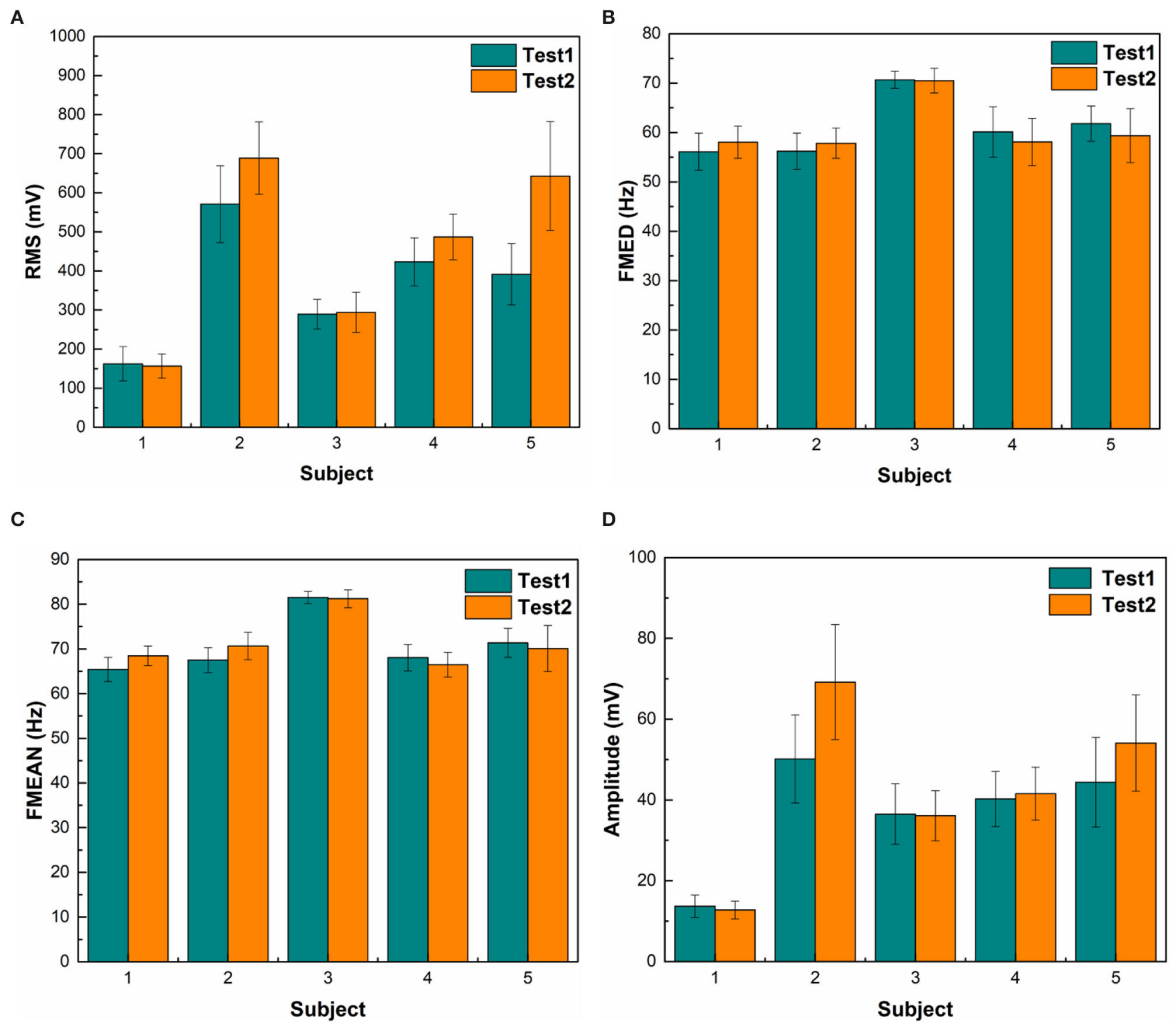
Statistical results of different features. **(A)** shows the EMG RMS of a muscle contraction. Green bars represent the first 20-repetition set of wrist motions and orange bars represent the second set of wrist motions. The RMS of Subject 1 is significantly lower than other subjects. RMS of the second set of wrist motions was higher than the first set in Subjects 2 and 5. **(B)** shows the median frequency (FMED) of a muscle contraction. There was not a significant change in FMED among subjects. **(C)** shows mean frequency (FMEAN). There was not a substantial change in FMEAN among subjects. **(D)** shows the mean magnitude of the 60–80 Hz bandwidth. The span of voltage difference of the 60–80 Hz band (57 mV) was smaller than that of RMS (532 mV).

The magnitude of RMS fluctuated among different subjects. The average RMS was 410.6 ± 187.9 (Mean ± SD). There was a statistical difference in EMG RMS between different subjects (ANOVA PostHoc LSD, *p* = 0.002). RMS was different between individuals (Subject 1 vs. Subjects 2, 3, 4 and 5: *p* = 0.000, 0.266, 0.007, and 0.003, respectively) (Table 1). The magnitude of RMS also fluctuated over time in individual subjects. There was a statistical difference in RMS between two sets of EMG data recorded at different time points in Subjects 2, 3, and 5 (*t*-test, *p* = 0.76, 0.04, 0.0002, 0.078, and 0.019 for Subjects 1 to 5, respectively) (Table 2). Different triggering thresholds were needed for each subject. A universal voltage value could not be used for all subjects, leading to the required onsite calibration for each subject (Figure 6A).

**Table 1:** Comparisons of measures among subjects during muscle contraction.

**Comparisons**	***P*** **-value**			
***ANOVA with PostHoc LSD***	**RMS**	**Magnitude of 60–80 Hz band**	**FMED**	**FMEAN**
Overall difference in subject groups	0.002	0.002	0.07	0.002
Subject 1 vs. subject 2	0.000	0.000	0.862	0.552
Subject 1 vs. subject 3	0.266	0.36	0.01	0.000
Subject 1 vs. subject 4	0.007	0.12	0.007	0.93
Subject 1 vs. subject 5	0.003	0.02	0.003	0.391

**Table 2:** Comparisons of measures between two time points during muscle contraction.

**Comparisons**	***P*** **-value**			
***T*-test**	**RMS**	**Magnitude of 60–80 Hz band**	**FMED**	**FMEAN**
Subject 1	0.76	0.69	0.715	0.824
Subject 2	0.04	0.095	0.444	0.656
Subject 3	0.0002	0.937	0.519	0.755
Subject 4	0.078	0.791	0.677	0.957
Subject 5	0.019	0.283	0.705	0.514

There was no significant change in FMED among Subjects 1, 2, 4, and 5 (ANOVA PostHoc LSD, *p* = 0.968), but Subject 3 had a significantly higher median frequency (ANOVA PostHoc LSD, *p* = 0.007) (Table 1). The FMED did not fluctuate over time in individual subjects. There was no statistical difference in FMED between the two sets of FMED data in the five subjects (*t*-test, *p* = 0.715, 0.444, 0.519, 0.677, and 0.705 for Subjects 1-5, respectively) (Table 2 and Figure 6B).

There was no significant changes in FMEAN among Subjects 1, 2, 4, and 5 (ANOVA PostHoc LSD, *p* = 0.756), but Subject 3 had a higher mean frequency than other subjects (*P* = 0.002) (Table 1). The FMEAN did not fluctuate over time in individual subjects. There was no statistical difference in the FMEAN between the two sets of the FMEAN data recorded at different time points in the five subjects (*t*-test, *p* = 0.824, 0.656, 0.755, 0.957, and 0.514 for Subjects 1-5, respectively) (Table 2). The prominent frequency band corresponding to muscle contraction was between 60 and 80 Hz. This frequency was the same bandwidth as FMED and FMEAN. The triggering threshold for the frequency bandwidth was not significantly adjusted for each subject and onsite calibration was not performed (Figure 6C).

The magnitude of the 60–80 Hz band also fluctuated among different subjects (Figure 6D). The average 60–80 Hz band voltage magnitude was 39.8 ± 17.1 (Mean ± SD). There was a statistical difference in the magnitude of the 60 to 80 Hz band between different subjects (ANOVA PostHoc LSD, *p* = 0.02). The magnitude of the 60–80 Hz band was different between individuals (Subject 1 vs. Subjects 2, 3, 4, and 5: *p* = 0.001, 0.36, 0.12, and 0.02, respectively) (Table 1). But different triggering thresholds were not needed for each subject. A universal voltage value could not be used for all subjects, leading to the required onsite calibration for each subject (Figure 6A). The magnitude of the 60–80 Hz band did not fluctuate over time in individual subjects evidenced by that there was not a statistical difference in the magnitude of the 60–80 Hz band between the two sets of EMG data recorded at different time periods (*t-*test, *p* = 0.69, 0.095, 0.937, 0.791, and 0.283 for Subjects 1–5, respectively) (Table 2). A universal voltage value was used for all subjects without onsite calibration.

Time-domain feature RMS shows greater variability among different subjects than the STFT-domain method (Figures 6A,D). There was a significant difference in the standard deviation (SD) between the magnitudes of RMS and the 60–80 Hz band (*t*-test, *p* = 0.0001). Even for the same subject, the RMS results between the two tests were different. The frequency-domain features were more stable between subjects. The average mean frequency of five subjects was 71.06 ± 5.43 Hz. Table 3 shows the details of EMG features. The statistical results show that the frequency domain feature was more suitable for EMG embedded control systems because of its stability.

**Table 3:** Statistical results of EMG feature selection experiment.

		**RMS (mV)**			**FMEAN (Hz)**			**FMED (Hz)**			**Magnitude (mV)** **60–80 Hz Band**		
		**Mean**	**Max**	**Min**	**Mean**	**Max**	**Min**	**Mean**	**Max**	**Min**	**Mean**	**Max**	**Min**
Sub 1	Test 1	162.4	292.2	112.5	65.4	69.3	59.8	56.1	62.6	47.8	13.6	23.5	10.2
	Test 2	156.6	249.6	119.4	68.4	77.6	65.5	58.0	64.9	52.1	12.7	16.7	9.4
Sub 2	Test 1	570.8	818.1	274.7	67.5	73.5	63.4	56.2	62.3	50.5	50.1	69.2	26.3
	Test 2	588.8	894.1	476.2	70.6	80.3	63.0	57.8	67.3	51.4	69.1	97.4	36.1
Sub 3	Test 1	289.3	354.8	212.9	81.5	83.9	77.1	70.7	73.5	66.5	36.5	48.9	21.2
	Test 2	293.9	393.4	218.4	81.2	87.7	77.1	70.5	75.3	66.7	36.1	43.4	24.4
Sub 4	Test 1	423.2	592.7	342.7	68.1	74.4	62.0	60.1	66.2	49.3	40.2	51.3	24.2
	Test 2	487.1	606.4	359.9	66.5	71.3	61.9	58.1	66.0	48.5	41.6	51.3	29.0
Sub 5	Test 1	391.5	564.8	198.9	71.3	78.8	64.8	61.8	71.3	53.6	44.3	63.4	24.3
	Test 2	642.5	930.4	302.6	70.1	78.4	60.4	59.4	59.4	48.1	54.1	70.9	24.9

### Accuracy of EMG-Based Embedded System Performance in Motion Control

Both RMS and STFT-based EMG processing embedded systems controlled robotic hand motion adaptively. The robotic hand moved or stopped at any time and at any position according to the user's intent. The average time delay of motion recognition was <300 ± 15 ms. The results demonstrated that the STFT frequency domain method was more suitable for EMG embedded control systems based on its performance stability. The frequency band of the 60–80 Hz was selected as the designated frequency band for triggering the motor motions depending on the magnitude of the voltage of this frequency band. When the amplitude of the 60–80 Hz band was higher than the threshold, the MCU sent the moving commanding pulse signal to the motor controller.

**Figure 7:**
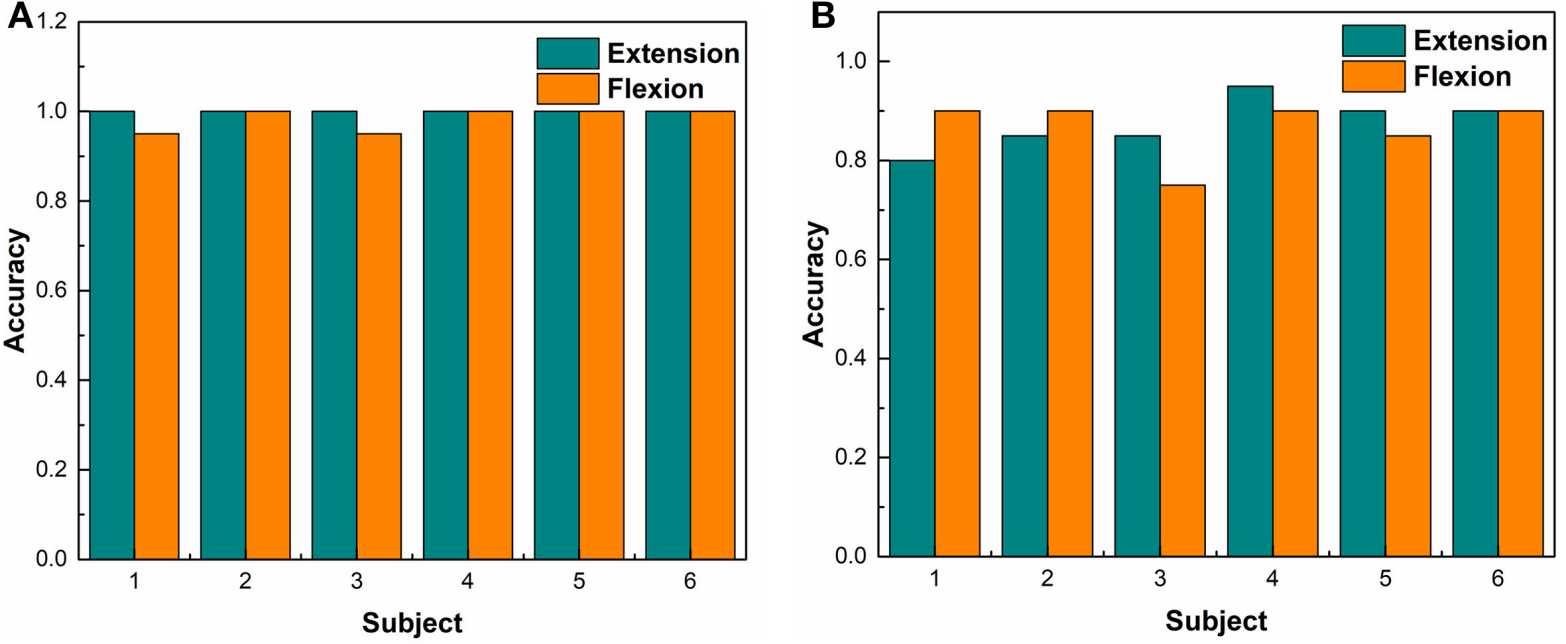
Accuracy of robotic hand control experiment among participants. **(A)** The results of RMS-based controlling algorithms. **(B)** The results of FFT – based controlling.

Figure 7A shows the results of using the time-domain features, which monitored all of the frequency band amplitude. For motor motion triggering, the accuracy of extension motion recognition was 100%, and the accuracy for flexion motion recognition was 98 ± 2%, while the accuracy for recognizing no motion was 99 ± 1%. However, the triggering threshold needed to be manually set up for each subject. Figure 7B shows the results of using the frequency-domain features, which monitored the EMG amplitude of the 60–80 Hz frequency band for motor triggering. The accuracy of flexion movement recognition was 90 ± 7% and the accuracy for recognizing extension movement was 86 ± 9%, while the accuracy for recognizing no movement was 96 ± 4%. The triggering threshold was pre-set and was not adjusted for different subjects. For the sensitivity of the system action, the average time delay of system response was <300 ms and the time delay was calculated from subject movements to the corresponding DC motors movements.

There was no statistical difference between the two processing methods (RMS vs. STFT processing method) (Chi-Square, Pearson test, *p* = 0.977) (Figure 6). However, more efforts were required for setting up triggering thresholds because there was more variability of RMS magnitude between subjects than the variance of the magnitude of the 60–80 Hz frequency band.

In terms of accuracy, it appeared to be that the RMS-based embedded system had a higher accuracy than the STFT embedded system, but there was no statistical difference. However, the variability of myoelectric signal magnitude was higher in RMS group that STFT group, leading to a significant amount of time required for pre-training when using the RMS-based embedded system. Moreover, the threshold set up and adjustment were required among different individuals upon using the RMS-based embedded system. There was no need of onsite calibration or threshold adjustment for STFT-based embedded system.

## Discussion

Myoelectric signals from electromyography (EMG) have been used for hand exoskeleton or assistive robotic device motion control for decades, yet there are still challenges to be solved (Da Silva et al., 2008; Ho et al., 2011; Ockenfeld et al., 2013; Song et al., 2013; Salvietti et al., 2016). The EMG-based signal processing methodology includes time-domain methods by monitoring the signal's voltage over time and frequency-domain methods by monitoring the frequency of the whole myoelectric signal spectrum. The time-domain RMS-based EMG signal processing method has been used for robot control as a popular approach for years, while frequency-domain methods including the STFT processing method have been proposed for many years, but no cost-effective small-size embedded system has been developed for a real-time robot motion control yet. Frequency-domain EMG processing for robot control utilized the whole myoelectric signal spectrum and most of the methods utilized machine learning (ML) methods for EMG signal processing (Englehart et al., 2001; Manal et al., 2002; Kuiken et al., 2009; Antuvan et al., 2016; Mastinu et al., 2018; Phinyomark et al., 2018; Zia ur Rehman et al., 2018; Yu et al., 2021).

Aiming at developing a cost-effective and wearable EMG signal processing for a robot control system, this study introduced a novel frequency-domain EMG signal processing embedded system for robot control. Instead of using the whole myoelectric signal spectrum, a fixed-bandwidth (60–80 Hz) frequency-domain EMG signal processing method was used for motion intent detection in this study. The results demonstrated that the fixed-bandwidth frequency-domain EMG processing method produced an alternated cost-effective approach for EMG signal processing and robot motion control. This frequency-domain method demonstrated a stable performance in robot control. The reason can be that focusing on a specific bandwidth of frequency associated with muscle contraction directly denoised the myoelectric signals.

Our goal is to develop an EMG-controlled robotic assistive device for industrial workers and clinical stroke survivors. Current work focuses on the applications in healthy subjects. Further research is required for the applications in stroke survivors. The myoelectric signals are weak in signal strength in stroke survivals, with a much lower signal-to-noise ratio (SNR). Abnormal muscle activation characteristics are found in stroke survivor associated with loss of dexterity after stroke (Canning et al., 2000). Fixed-bandwidth frequency-domain EMG processing method may provide an optimal method by focusing on a specific bandwidth of frequency associated with muscle contraction among stroke survivors.

Time-domain RMS-based methods monitor the magnitude of myoelectric signal voltage over time for signal processing and robot control. This approach has been reported in the literature with many technical challenges to be solved (Da Silva et al., 2008; Ho et al., 2011; Ockenfeld et al., 2013; Song et al., 2013; Salvietti et al., 2016; Phinyomark et al., 2018). As shown in this study, the voltage magnitude of the EMG signals in the RMS-domain fluctuated among different individuals resulting in more variance in average voltage readings, leading to no universal threshold that can be set up to trigger DC motor motion. This led to a requirement of onsite calibration to set up the triggering threshold for different users upon using the RMS-based embedded system. Moreover, noise and artifacts could trigger unwanted robot motion, hence reducing system performance accuracy.

Frequency-domain features and short-time Fourier transforms (STFT) have been used for spectral analysis of EMG signals (Englehart et al., 2001; Phinyomark et al., 2018). However, these previous studies mainly used machine learning (ML) for frequency-domain features processing (Da Silva et al., 2008; Larivière et al., 2008; Camata et al., 2010; Costa et al., 2010; Dantas et al., 2010) and most of these studies were offline data analysis for conceptualization rather than real-time signal processing for instant robot motion control. The support vector machine (SVM) and CNN are two popular ML methods used for ML-based EMG signal processing. Only a few studies successfully demonstrated the ML approaches to process EMG signals for real-time robot control using both RMS and frequency variables (Zhou et al., 2021). A powerful computer and LabView software with a machine learning toolbox are required to implement the ML tasks with significant efforts devoted to establishing portal communication between EMG sensor systems and ML processing toolbox software. The study demonstrated that ML methodology and approaches cannot deliver a lightweight, cost-effective, wearable embedded system yet for EMG signal processing and control currently (Zhou et al., 2021). In addition, many ML algorithms have been proposed for EMG signal processing but mainly use pattern recognition (PR) for trajectory movement control (Da Silva et al., 2008; Larivière et al., 2008; Camata et al., 2010; Costa et al., 2010; Dantas et al., 2010; Jiang et al., 2020; Zhou et al., 2021). According to our own experience in processing 12-channel shoulder EMG signals using ML-based pattern recognition for upper limb exoskeleton control (Jiang et al., 2020; Zhou et al., 2021), a laptop and complex algorithms (LabView with ML toolbox) were required instead of a simple embedded hardware. A large amount of data was needed to be collected for model training. Significant efforts were required to connect Delsys EMG sensors, computer, NI data board, and upper limb exoskeleton. Multiple factors, such as motion speeds, device difference, and individual variability, affected the accuracy (ranging from 75 to 97%) of system performance (Jiang et al., 2020). These studies demonstrated that ML can be used successfully for trajectory movement control based on pattern recognition, but processing EMG signals using ML for adaptive robot movement control needed further investigation. Fortunately, this current study demonstrated that cost-effective embedded system using MCU and the fixed bandwidth STFT algorithms can be used to implement one DoF of adaptive motion control.

Studies have shown that cost-effective EMG sensors have been reported for EMG-controlled robotic assistive systems. Myo-armband EMG sensor has a sample rate of 200 Hz, which causes loss of higher-frequency content (>100 Hz), but still captured muscle contraction signal between 50 and 100 Hz (Phinyomark et al., 2018). In our study, the MyoWave^TM^ sensor is also an inexpensive EMG sensor. It was used in this study and did not cause a loss in higher-frequency content. Moreover, the magnitude of the frequency band between 60 and 80 Hz was captured during muscle contraction. This feature enabled microcontroller units (MCU), such as STM32, to implement the STFT algorithm focusing on monitoring the magnitude of interested bandwidth for motion classification.

STM32 is a simple, inexpensive, small, embedded hardware with a capacity to implement frequency-domain (FFT) signal processing and real-time commanding signal outputs for DC motor control. The voltage magnitudes of different frequency bandwidths between 1 and 500 Hz were clearly shown using STM32 and its IDE software, leading to a readily preparation of encoding algorithms. The mean frequency (71 Hz) of the EMG signal correlated to a muscle contraction. Hence, the 60–80 Hz frequency band was selected for EMG signal processing in this study. Algorithms were encoded to monitor the magnitude of the 60–80 Hz frequency bandwidth for motion classification. Using this fixed-bandwidth frequency-domain voltage threshold set up to trigger the DC motor avoided extra efforts and procedures for signal denoising. The average time delay of the motion control system was <300 ms and the average recognition accuracy of motor control was 91.55%. This method presents a novel approach for developing a less complex embedded EMG processing system for robot motion control.

In terms of a population-level standard of using 60–80 Hz for STFT processing, the frequency range of 60–80 Hz appeared to be a reasonable standard for ~95% of the population. In this study, the mean frequency was 71.0 ± 5.7 (1 SD) Hz. When the frequency range was 71.0 ± 11.4 (2 SD) Hz, it was approximated within the range of 60–80 Hz. Two-standard deviations (SDs) cover 95% of cases statistically (Pukelsheim, 1994).

A comparison of performance efficiency between time-domain and frequency-domain methods was performed in this study. Currently, time-domain methods are commonly used for EMG signal processing and control (Raurale et al., 2019; Secciani et al., 2019), but onsite calibration was also required in this study. The studies using time-domain features (Raurale et al., 2019; Secciani et al., 2019) required a significant amount of time to train a person to identify the optimal setup for machine operation. EMG amplitude fluctuation was a common issue leading to a difficult setup for a triggering threshold for DC motor control. In our study, although the system using time-domain features achieved high accuracy, a significant amount of time was also spent for different subjects to train the system for optimal setup, which was the same as reported in the literature. Using sensor fusion-based myoelectrical control (Su et al., 2021b; Qi and Su, 2022) is a future direction, but these methods usually require a high computational power which is difficult for embedded systems to achieve.

When using the fixed bandwidth frequency-domain features, the EMG voltage within the 60–80 Hz frequency bandwidth had less deviation during muscle contraction, resulting in less effort for system set up and training time. The fixed bandwidth frequency-domain algorithms produced smoother exoskeleton motions and did not need onsite calibration. The amplitude of a fixed frequency band had less fluctuation of EMG voltage.

The limitation of this study is that a clinical study has not been performed among patients. We assume this system can detect weak remnant EMG signals in stroke patients but have not tested the embedded system in clinical settings. Clinical trials will be performed for robotic assistive devices. In this study, only one degree of freedom (DoF) motion control was developed, the feasibility of the embedded system for multiple DoFs of robot control needs to be investigated in the future.

## Conclusion

In this study, a real-time fixed bandwidth frequency-domain EMG-based controlling system for the exoskeleton was developed. The system showed better sensitivity and stability in recognizing muscle contraction than the time-domain features-based system. The average time delay of motion recognition was <300 ms and the average accuracy of motor control was 91.55%. This study also found that EMG amplitude of a spectrum band between 60 and 80 Hz predominantly responded to muscle contraction. Using EMG signal amplitude of this frequency band to set up the DC motor motion triggering threshold was a feasible and reliable approach and spared the extra effort required for systematic noise removal.

## Data Availability Statement

The raw data supporting the conclusions of this article will be made available by the authors, without undue reservation.

## Ethics Statement

The studies involving human participants were reviewed and approved by the Institutional Review Board (IRB) of Wayne State University (#1905002258). The participants provided their written informed consent to participate in this study.

## Author Contributions

BC and CC contributed to the conceptualization, methodology, validation, and wrote the original draft of the manuscript. JH, TN, JQ, BY, and DC contributed to the investigation, formal analysis, and system debugging. YA-r, YZ, AT, TF, and HG contributed to the writing—review and editing. All authors have read and agreed to the published version of the manuscript.

## Funding

This research was supported by the National Natural Science Foundation of China (51975360 and 52035007), the National Social Science Foundation of China (17ZDA020), and the Cross Fund for medical and Engineering of Shanghai Jiao Tong.

## Conflict of Interest

The authors declare that the research was conducted in the absence of any commercial or financial relationships that could be construed as a potential conflict of interest.

## Publisher's Note

All claims expressed in this article are solely those of the authors and do not necessarily represent those of their affiliated organizations, or those of the publisher, the editors and the reviewers. Any product that may be evaluated in this article, or claim that may be made by its manufacturer, is not guaranteed or endorsed by the publisher.
